# Tissue Regeneration in Dentistry

**DOI:** 10.1155/2012/586701

**Published:** 2012-05-15

**Authors:** Kazuo Tanne, Petros Papagerakis, Gianpaolo Papaccio, Chiaki Kitamura, Kotaro Tanimoto

**Affiliations:** ^1^Department of Orthodontics and Craniofacial Developmental Biology, Hiroshima University Graduate School of Biomedical Sciences, Hiroshima 734-8553, Japan; ^2^Department of Orthodontics and Pediatric Dentistry, Center for Organogenesis, Center for Computational Medicine and Bioinformatics, Schools of Dentiry and Medicine, University of Michigan, Ann Arbor, MI 40109, USA; ^3^Department of Histology and Embryology, DIMES-Histology, Tissue Engineering and Regenerative Medicine Laboratory (TERM), Secondo Ateneo di Napoli, 5, Via L. Armanni, 80138 Napoli, Italy; ^4^Department of Pulp Biology, Operative Dentistry, and Endodontics, Kyushu Dental College, 2-6-1 Manazuru, Kokurakita, Kitakyushu 803–8580, Japan; ^5^Department of Orthodontics, Hiroshima University Hospital, Hiroshima 734-8553, Japan

Recently, various studies for tissue engineering have been conducted successfully to explore a new era of tissue defect treatment. To this end, three essential factors were examined extensively, that is, cells to be transplanted, signaling molecules, and scaffold. Currently, undifferentiated mesenchymal stem cells (MSCs) or the differentiated forms are transplanted with the appropriate scaffolds, producing excellent tissue regeneration. In addition, it is demonstrated that various signaling molecules such as basic FGF, BDNF, BMP, and TGF-beta are available for new tissue formation.

Under such background, a special issue on tissue regeneration in dentistry was designed to summarize the current status of tissue regeneration. Thus, we have invited authors to submit original research and review articles that seek the nature of tissue regeneration and the relevant factors. As the results, various interesting and scientifically significant papers have been accepted. These studies are categorized into MSCs and/or dental MSCs and the relevant factors, cellular responses to various signaling molecules and biomaterials,* in vivo* studies for tissue regeneration in animal models with artificially created tissue defects, and confirmation for the safety of tissue regeneration with ethical consideration.

